# Roles for B[*a*]P and FICZ in subchondral bone metabolism and experimental temporomandibular joint osteoarthritis via the AhR/Cyp1a1 signaling axis

**DOI:** 10.1038/s41598-021-94470-4

**Published:** 2021-07-21

**Authors:** Yuri Yoshikawa, Takashi Izawa, Yusaku Hamada, Hiroko Takenaga, Ziyi Wang, Naozumi Ishimaru, Hiroshi Kamioka

**Affiliations:** 1 Department of Orthodontics, Graduate School of Medicine, Dentistry and Pharmaceutical Sciences, Okayama University, 2-5-1 Shikata-cho, Kita-ku, Okayama, 700-8525 Japan; 2 Department of Oral Molecular Pathology, Tokushima University Graduate School of Biomedical Sciences, 3-18-15 Kuramoto-cho, Tokushima, 770-8504 Japan

**Keywords:** Cell death, Osteoimmunology, Inflammation, Experimental models of disease, Biochemistry, Cytokines, Histocytochemistry

## Abstract

Bone loss due to smoking represents a major risk factor for fractures and bone osteoporosis. Signaling through the aryl hydrocarbon receptor (AhR) and its ligands contributes to both bone homeostasis and inflammatory diseases. It remains unclear whether the same AhR signaling axis affects the temporomandibular joint (TMJ). The aim of this study was to investigate possible mechanisms which mediate bone loss in the TMJ due to smoking. In particular, whether benzo[*a*]pyrene (B[*a*]P), a carcinogen of tobacco smoke, induces expression of the AhR target gene, Cyp1a1, in mandibular condyles. Possible functions of an endogenous ligand of FICZ, were also investigated in a TMJ-osteoarthritis (OA) mouse model. B[*a*]P was administered orally to wild-type and *AhR*^−/−^ mice and bone metabolism was subsequently examined. TMJ-OA was induced in wild-type mice with forceful opening of the mouth. Therapeutic functions of FICZ were detected with μCT and histology. Exposure to B[*a*]P accelerated bone loss in the mandibular subchondral bone. This bone loss manifested with osteoclastic bone resorption and upregulated expression of Cyp1a1 in an *AhR*-dependent manner. In a mouse model of TMJ-OA, FICZ exhibited a dose-dependent rescue of mandibular subchondral bone loss by repressing osteoclast activity. Meanwhile, in vitro, pre-treatment with FICZ reduced RANKL-mediated osteoclastogenesis. B[*a*]P regulates mandibular subchondral bone metabolism via the Cyp1a1. The AhR ligand, FICZ, can prevent TMJ-OA by regulating osteoclast differentiation.

## Introduction

A long-term smoking habit can have many consequences, one is the development of osteoporosis. This condition is recognized as a critical determinant of fracture risk. For those with a history of smoking, there is more than 85% high risk factor of hip-fracture and up to a 40% higher overall risk factor of fracture^1,2^. The mechanistic details mediating these effects are unknown, especially in relation to metabolism of the mandibular subchondral bone. Subchondral bone and articular cartilage act as a unit to maintain the structural and functional integrity of joint^3,4^. Subchondral bone provides the mechanical support and nutrition supply for overlying articular cartilage^5^. The presence of more than 4000 compounds in cigarette smoke also represents a daunting number of candidates to consider.

Signaling through the aryl hydrocarbon receptor (AhR) and its ligands has been shown to contribute to both bone homeostasis and inflammatory diseases. The effects of halogenated and polycyclic aromatic hydrocarbons (PAH) in the environment, including benzo[*a*]pyrene (B[*a*]P) containing in cigarette smoke, are also mediated through the AhR. It has been established that binding of agonist B[*a*]P to the receptor AhR induces translocation of the complex to the nucleus where it forms a heterodimer with AhR nuclear translocator (ARNT). As a basic helix-loop-helix transcription factor of the Per–Arnt–Sim (PAS) family^6^, this heterodimer is able to bind xenobiotic response elements present in the promoter regions of target genes including cytochrome P4501A1 (Cyp1a1)^7^. Cyp1a1 is an enzyme that is specifically trans-activated through AhR signaling axis and has been implicated in the bio-activation via B[*a*]P. We previously demonstrated that the c-Fos-AhR transcription factor complex plays an crucial role in regulating osteoclast differentiation^8^.

The incidence of temporomandibular joint osteoarthritis (TMJ-OA) has been increasing over the past decade. While many hypotheses have been proposed regarding the etiology of this multi-pathogenesis disease, the mechanistic details of its pathological progression remain unclear. To investigate the pathogenesis of TMJ-OA, various animal models have been established. These models employ mechanical force, surgery, and chemical or transgenic methods. Subchondral bone disturbance has been identified as a key element with the progression of OA as well as overlying cartilage degeneration^9^. We have reported spontaneous abnormalities in the mandibular condyle subchondral bone can induce progressive cartilage degradation in mice^10^. It was also reported that overexpression of TGF-β1 in subchondral bone can induce progressive cartilage degradation in mice^11^. When excessive mechanical loading is received into healthy articular cartilages, or when physiologic mechanical loading forces is applied to pathologic articular cartilage, these cases can induce osteoarthritis^12–14^. We previously reported that the continuous and compressive mechanical loading force can induce a series of patho-physiological features and changes within mandibular articular cartilage, including reduced cartilage elasticity, extracellular-matrix degradation, and chondrocyte death^15^. These pathological changes can also be mediated by programmed cell death and endoplasmic reticulum stress^13,16^, while other mechanisms and therapy may also be relevant.

Endogenous natural ligands of AhR include flavonoids and indoles. In particular, 6-formylindolo[3,2-b]carbazole (FICZ) is a high-affinity endogenous ligand partner with nuclear receptor AhR which has been widely studied in both in vitro and in vivo inflammatory disease models^17^. FICZ is generated from tryptophan metabolism following exposure to ultraviolet or visible light. Correspondingly, FICZ is physiologically relevant in human skin^18,19^. Here, we tested the hypothesis that expression of Cyp1a1 is disrupted in TMJ-OA, and the AhR ligand, FICZ, may provide protection. Histomorphological and micro-CT analyses were performed, as well as immunohistochemistry (IHC) and real-time qPCR to detect levels of a representative AhR target gene, Cyp1a1, and osteoclastic markers, cathepsin K, integrin β_3_, and tartrate-resistant acid phosphatase (TRAP).

## Materials and methods

### Mouse models

Specific pathogen-free mouse colonies of C57BL/6 mice (Japan SLC Laboratory, Shizuoka, Japan) as a control and *AhR*^−/−^ mice [RIKEN BioResource Research Center (RBRC02976)] were maintained. Water and food were provided ad libitum. Experiments were humanely conducted and were approved by the Animal Care and Ethical & Use Committee (Tokushima University, Japan, Permit No. toku-09021). Animal experiments were carried out in compliance with the ARRIVE guidelines (https://arriveguidelines.org) and the NIH guidelines (Guide for the Care and Use of Laboratory Animals).

### Treatments and TMJ-OA model

Both WT and *AhR*^−/−^mice were administered B[*a*]P (120 mg/kg; Tokyo Chemical Industry, Tokyo, Japan) or corn oil (control) daily via oral gavage in six doses for 6 days.

WT mice were subjected to forced mouth opening-induced TMJ-OA^15^ and received a tail vein injection of FICZ twice a week. Two different concentrations of FICZ were administered (FICZ low: 100 μg/kg, FICZ high: 100 mg/kg). FICZ (Focus Biomolecules #10-1463, Plymouth Meeting, PA, USA) was dissolved in dimethyl sulphoxide (DMSO) (0.5 mg/mL) for these injections. Experimental TMJ-OA was established according to a forced mouth opening method as previously described^15^. In brief following an intraperitoneal injection of 50 mg/kg somnopentyl, adverse mechanical stress was applied to the TMJ of mice with a consistent and repetitive mouth-opening protocol. A custom-made spring was used to deliver a force of 2N at maximal mouth opening (measured to be 14 mm, passively, in 8-week-old C57BL/6 WT mice). The TMJ of the mice in the loaded group was subjected to mechanical loading by forceful opening of the mouth for 3 h/days for 5 days. Individual spring forces were measured with a mechanical test system (autograph AG-X 1 kN, SHIMADZU, Kyoto, Japan). This method was approved by the Animal Care and Ethical & Use Committee of Okayama University (Permit No. OKU-2020861).

### Isolation of macrophage and primary mandibular chondrocytes

Bone marrow macrophages (BMMs) were differentiated into multinucleated mature osteoclasts as previously described^20^. Briefly, isolated BMMs were cultured with macrophage colony stimulating factor (M-CSF, 20 ng/mL) and receptor activator of nuclear factor kappa-B ligand (RANKL, 100 ng/mL) for 6 days. TRAP activity was subsequently detected with staining (Kit 387-A; Sigma-Aldrich, St. Louis, MO, USA). For applications in vitro*,* FICZ was dissolved in DMSO and prepared at concentrations of 10, 100, or 200 ng/mL. Primary mandibular chondrocyte cells were isolated from the condyles according to a previously published procedure^10^. In brief, TMJ condylar cartilage tissues were dissected from 6 to 10-week-old mice. Pieces of cartilage were minced with a scalpel and then were digested with 3 mg/mL collagenase (FUJIFILM Wako Pure Chemical, Osaka, Japan) and 4 mg/mL dispase (Gibco, Grand Island, NY) in PBS at 37 ℃ with shaking. After 3 h, enzymatic digestion was stopped. The resulting cell suspension was filtered through a nylon mesh (70-μm pore size; BD Falcon, Franklin Lakes, NJ) to eliminate cell–matrix residues, then was centrifuged for 10 min at 250×*g*. The chondrocytes obtained were washed thrice with α-MEM and cultured in 5% CO_2_ at 37 ℃ in basal medium that consisted of α-MEM supplemented with 20% FBS, 2 nmol/L glutamine, 100 U/mL penicillin, 100 mg/mL streptomycin (Gibco), and 100 mmol/L 2-mercaptoethanol (Gibco). After 4–6 days, the adherent cells were detached with trypsin–EDTA (Gibco) and were passaged.

A mouse chondroprogenitor cell line, ATDC5 (RIKEN BioResource Center Cell Bank, Tsukuba, Japan), was cultured as a monolayer in high-glucose Dulbecco’s modified Eagle’s medium (Sigma-Aldrich) with 5% FBS.

### μ-Computed tomography (μCT)

Mandibular condyles were resected from wild-type (WT) and *AhR*^−/−^ mice treated with B[*a*]P. After soft tissues were removed, the bones were incubated in 70% ethanol overnight. Image acquisition was performed the next day with a high-resolution microcomputed tomography scanner (SkyScan 1176 Scanner; Bruker, Billerica, MA, USA) at 200 μA and 50 kV. To prevent dehydration and movement, samples were tightly covered with plastic wrap during image acquisition. Thresholding was applied to distinguish background noise from bone images. A posterior region in the midsagittal section of the mandibular condyle was established as the region of interest (ROI). At a resolution of 9 μm/pixel, microstructural parameters of each ROI were analyzed. These parameters included: trabecular separation (Tb.Sp), trabecular thickness (Tb.Th), and bone volume to trabecular bone volume ratio (BV/TV).

### Tissue preparation, histologic staining, and analysis

Resected TMJ tissues were fixed in freshly prepared 4% paraformaldehyde (PFA)/phosphate-buffered saline (PBS) for 2 days and decalcified in the EDTA for 20 days and then embedded into paraffin. After preparing serial sagittal sections using a microtome (Carl Zeiss HM360, Jena, Germany), sections were stained with TRAP in order to detect multinucleated-osteoclasts, while staining with Hematoxylin–Eosin (HE) and Safranin-O provided scoring for features of cartilage disease (e.g., structural abnormalities and changes in cellularity). Glycosaminoglycan loss and distribution were also examined. In brief, area of proteoglycan staining of the cartilage of the central and posterior thirds of the mandibular condyles were quantified using Image J software (NIH, Bethesda, MD, USA)^21^. A modified Mankin scoring system was used to assess the degree of cartilage degeneration^22^. Safranin-O-stained sections were used to score samples for features of cartilage damage, including changes in cellularity, structural abnormalities, and uptake of safranin-O as a measure of glycosaminoglycan distribution and loss. Experts (TI, NI, and HK) were independently blinded to the identity of the samples during the scoring and analysis of sections.

### IHC

Paraffin sections were subjected to a gradient of ethanols for deparaffinization and then were blocked with 5% (v/v) bovine serum albumin (BSA) for a half hour. The sections were subsequently reacted with rabbit polyclonal antibodies diluted in 0.1% BSA/PBS which recognize: Cyp1a1 (sc-20772; Santa Cruz Biotechnology, Dallas, TX, USA), aggrecan (ab36861; Abcam, Cambridge, United Kingdom), Col2a1 (ab21291; Abcam), Sox9 (ab3697; Abcam), cleaved caspase-3 (#9661; Cell Signaling Technology, Danvers, MA, USA), and AhR (BML-SA210; ENZO Life Sciences, Farmingdale, NY, USA). After an incubation step kept in 4 °C overnight, the sections were washed several times by PBS and then reacted with secondary IgG antibodies as appropriate. After 1 h at room temperature, bound antibody complexes were visualized with 3,3-diaminobenzidine (DAB) at 2.5 mg/mL and counterstained with 0.1% methyl green. Stained sections were mounted and observed using the BioRevo BZ-9000 microscope and analyzing softwares (KEYENCE, Osaka, Japan).

### Detection of apoptotic cell death by TdT-mediated dUTP-digoxigenin nick-end labeling (TUNEL) staining

A TUNEL method was used to assay the distribution of apoptotic chondrocyte cells. After the 3′-hydroxyl terminus of DNA strand breaks were labeled, apoptotic cells were detected using an In Situ Apotosis Detection Kit (FUJIFILM Wako Pure Chemical), according to the attached company’s protocol. Microscopy was used to observe TUNEL^+^ apoptotic cells (KEYENCE).

### Isolation of RNA

Frozen condyles were homogenized with Precellys Evolution lysis and homogenization system (Bertin Instruments, Montigny-le-Bretonneux, France). A PureLink RNA isolation mini kit (Thermo Fisher Scientific, Waltham, MA, USA) was subsequently used in order to isolate mRNA, followed by the manufacturer’s instructions. The NanoDrop One spectrophotometer (Thermo Fisher Scientific) was utilized to estimate RNA concentrations.

### Quantitative PCR (q-PCR)

Total RNA was extracted using PureLink spin column kit (Thermo Fisher Scientific), and a ReverTra Ace qPCR RT kits (TOYOBO, Osaka, Japan) were used to reverse transcribe mRNA into cDNA, according to the company’s instructions. Each cDNA samples were then prepared in a 10-μl total volume qPCR reaction containing real-time PCR Master Mix SYBR Green (TOYOBO) and 1 μM primer pairs. Levels of *Trap*, *Cathepsin K*, *Integrin β*_3_, *Cyp1a1*, *aggrecan*, *Col2a1*, and *Sox9* mRNA were analyzed with a LightCycler 96 System (Roche Diagnostic, Mannheim, Germany). These levels were corrected by the glyceraldehyde 3-phosphate dehydrogenase, GAPDH, as an endogenous control and then evaluated according to the comparative cycle threshold method (ΔΔCt). The primer sequences used in amplification are shown in Supplemental Table S1.

### Western blotting analysis

Lysis of cells was performed with RIPA lysis buffer containing freshly prepared Halt protease (0.1%) inhibitor cocktail (Thermo Fisher Scientific). Forty micrograms of each sample per 1 lane [detected with a BCA Protein Assay kit (Thermo Fisher Scientific)] were electrophoretically fractionated with SDS-PAGEs of appropriate percentages and then transferred to polyvinylidene (PVDF)-membranes. After an overnight incubation in 5% skim milk/TBS-T kept at 4 °C, the transferred membranes were reacted with first antibody recognizing: Cyp1A1; sc-20772, NFATc1; sc-7294 (Santa Cruz Biotechnology), caspase-3; #9662, cleaved caspase-9; #9509, integrin β_3_; #4702 (Cell Signaling Technology), c-Src (ab16885; Abcam), or Cathepsin K (C8243; Sigma-Aldrich), as appropriate. Beta-actin antibody was also included to provide a loading control (A5441; Sigma-Aldrich). After an overnight incubation, the membranes were well washed and then further reacted with horseradish-peroxidase-conjugated anti-mouse second antibody (AP124P; Millipore, Billerica, MA, USA) or anti-rabbit secondary antibodies (#7074; Cell Signaling Technology) at 20 °C. After 1 h, bound antibodies were detected with the LumiGLO Western Blot Detection System (Cell Signaling Technology).

### Staining of actin ring and bone resorption pits

BMMs were plated on dentin slices and cultured with M-CSF and RANKL for 6 days to generate mature osteoclasts. Dentin slices were fixed with 4% PFA in PBS for 20 min and permeabilized in 0.1% Triton X-100. F-actin was stained with Alexa Fluor 488-phalloidin. The number of osteoclasts with actin rings were counted per dentin slice under a conventional microscope equipped with a charge-coupled device camera (Olympus, Tokyo, Japan). The cells were then removed from dentin slices with mechanical agitation. Dentin slices were incubated with peroxidase-conjugated wheat germ agglutinin (Sigma Aldrich) for 1 h and stained with 3,3′-diaminobenzidine (Sigma Aldrich) for 30 min. Bone resorption pits were analyzed using a light microscope (KEYENCE) and pits area per field were quantified using Image J software (NIH)^21^.

### Analysis of osteoblast mineralization and differentiation

MC3T3 E1 cells were grown up to 21 days in osteogenic media including 20 mM β-glycerophosphates and 50 mM ascorbic acids. After fixation cells were appropriately stained using 0.2% alizarin-red S and alkaline phosphatase staining kit (AK20; Cosmo Bio, Sapporo, Japan). Osteoblastic marker genes such as *Alpl*, *Osteocalcin*, and *Col1a1* were detected by qPCR. Osteoblastic activities were analyzed in tetracycline and calcein double-labeled sections in vivo.

### Statistical analysis

Each experiment was independently repeated more than three times; and for the all sets of each condition, experiments were performed at least in duplicate or triplicate. Statistical data analyses were respectively performed by applying one-way analysis of variance (ANOVA) accompanied with post-hoc Tukey’s honest significant differences test or Student *t*-test, as appropriate for each case. *p* values less than 0.01 or 0.05 were considered as statistically significant.

## Results

### AhR is required for B[*a*]P-induced resorption of mandibular subchondral bone

**Figure 1:**
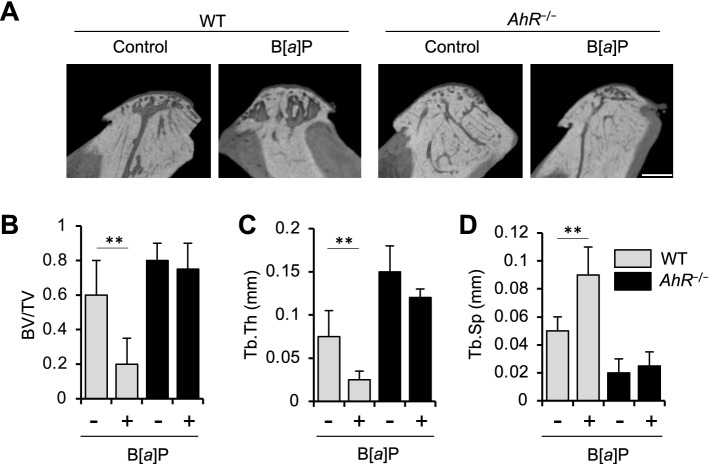
Analyses in vivo imaging of the condylar heads resected from mandibules of B[*a*]P-administered mice subjected to micro-CT. (**A**) Representative 3D sagittal section views from μCT scannings of mandibular condyles from WT and *AhR*^−/−^ mice administered with B[*a*]P or corn oil itself (control). Scale white bar = 500 μm. (**B**–**D**) bone volume/trabecular volume, BV/TV (**B**), trabecular thickness, Tb.Th (**C**), and trabecular separation, Tb.Sp (**D**). Data are presented as the mean ± SD (n = 7 mice per group). **p < 0.01.

Both WT and *AhR*^−/−^ mice received six oral doses of B[*a*]P (120 mg/kg daily) for six days. Trabecular thickness and BV/TV were subsequently evaluated with micro-CT. Significantly greater trabecular separation was observed in the B[*a*]P-treated WT mice than in the corn oil-treated control WT mice (Fig. 1A–D). In addition, trabecular thickness was reduced in different several regions of the subchondral bones manidibular condyles in the B[*a*]P-treated WT mice, while mandibular condylar bone mass in the *AhR*^−/−^ mice treated with B[*a*]P was unaffected (Fig. 1A–D).

### AhR ligand B[*a*]P increases osteoclastic activity

**Figure 2:**
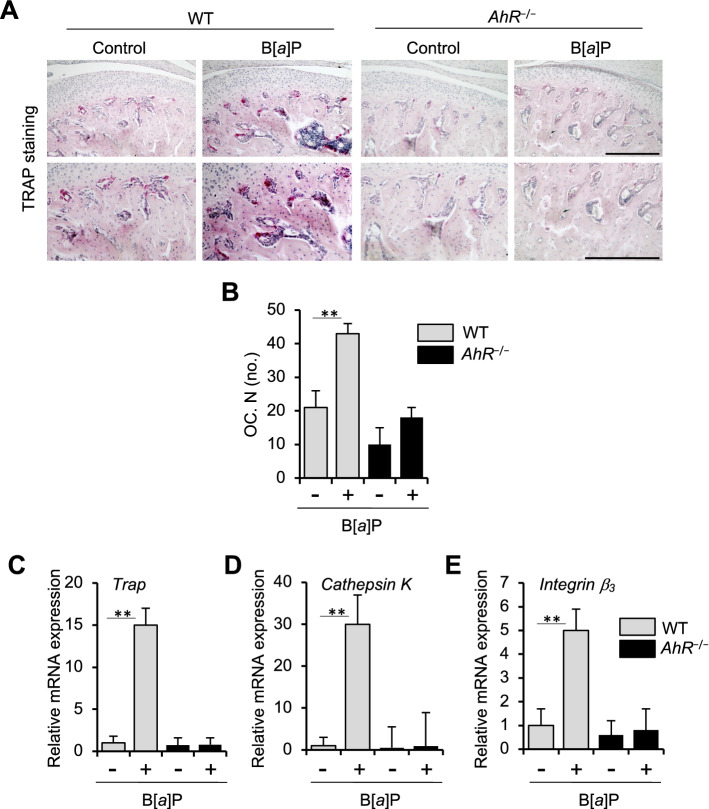
Numbers of osteoclasts with TRAP^+^ multinuclear in the mandibular condyles subchondral bone of WT and *AhR*^−/−^ mice treated with B[*a*]P. Representative sections of mouse mandibular condylar subchondral bone from corn oil- (control) or B[*a*]P-treated (oral gavage, 120 mg/kg) WT and *AhR*^−/−^ mice are shown. (**A**) TRAP staining. Scale bars = 300 μm. (**B**) Quantitative in vivo analyses of TRAP^+^ cells/mm bone perimeter. Mean ± SD (n = 6 mice/group). (**C**–**E**) Expression of *Trap* (**C**), *Cathepsin K* (**D**), and *Integrin β*_3_ (**E**). **p < 0.01.

To examine whether osteoclastogenic activity is affected by B[*a*]P in vivo, TRAP staining was performed. Compared with corn oil-treated control WT mice, osteoclast activity was significantly accelerated in B[*a*]P-administered WT mice (Fig. 2A,B). It was previously demonstrated that the femoral metaphysial trabecular bone of *AhR*^−/−^ mice exhibited an increased bone mass compared with WT controls^23^. Consistent with this report, we also observed an increase in bone mass in the mandibular condylar subchondral regions in *AhR*^−/−^ mice. Meanwhile, TRAP activity in the same bone was unaffected by oral administration of AhR ligand-B[*a*]P (Fig. 2A,B). Mandibular condyles from the B[*a*]P oral administered WT mice also had markedly higher mRNA levels of *Trap*, *Cathepsin K*, and *Integrin β*_3_, consistent with the higher osteoclastogenic activity detected in vivo. In contrast, these mRNA levels were suppressed in mandibular condyles from B[*a*]P-treated *AhR*^−/−^ mice (Fig. 2C–E).

### Cyp1a1 and caspase expression is necessary for B[*a*]P-induced mandibular bone resorption

**Figure 3:**
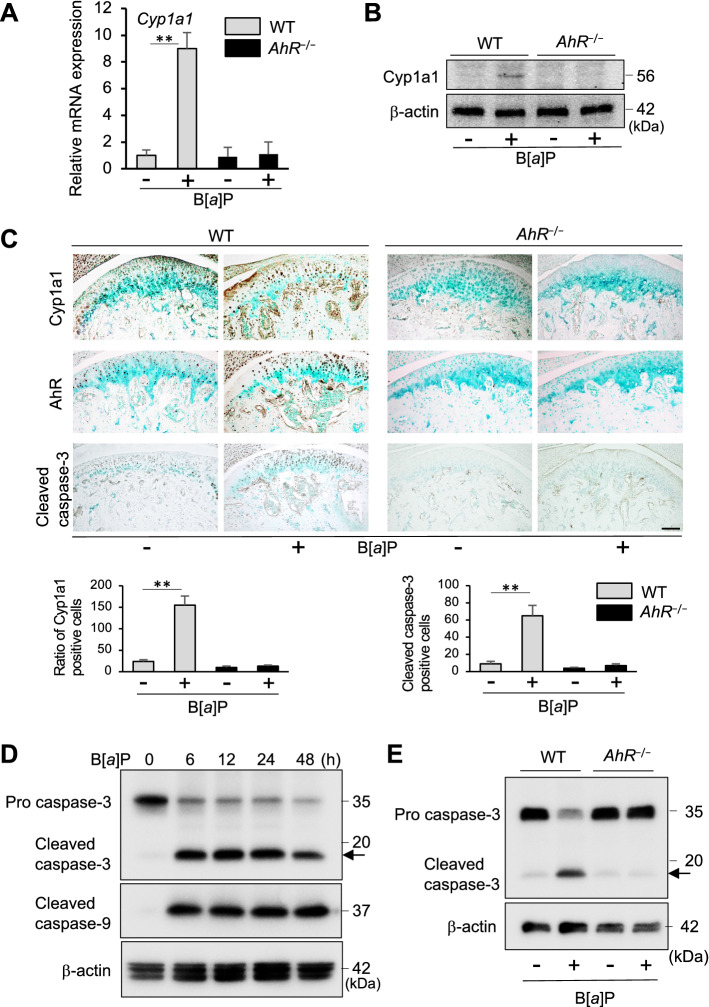
AhR is necessary for Cyp1a1 induction and caspase-3 cascade activation in B[*a*]P-treated mandibular condyles. (**A**,**B**) Detection of *Cyp1a1* mRNA (**A**) and protein (**B**) in mandibular condyles from WT and *AhR*^−/−^ mice treated with B[*a*]P (+ , oral gavage, 120 mg/kg) by qPCR and Western blotting, respectively. Staining by β-actin antibody was performed as a control of same amount protein loading. Full-length blots are presented in Supplementary Figure S2. (**C**) Representative sections from performing IHC staining to detect Cyp1a1, AhR and caspase-3 protein expression in mandibular condyles from WT and *AhR*^−/−^ mice treated with B[*a*]P (+ , oral gavage, 120 mg/kg). Scale bar = 100 μm. The number of Cyp1a1^+^ and active caspase-3^+^ cells in the posterior thirds and central of the mandibular condylar cartilages resected from *AhR*^−/−^ and WT mice administered with B[*a*]P were compared. (**D**) ATDC5 cells were incubated in the presence of 10 μM concentration B[*a*]P for 6, 12, 24, 48 h. Whole cell lysates were respectively subjected to immunoblot analyses to detect the apoptotic key marker cleaved caspase-3. Detection by β-actin antibody was performed as a control of same amount protein loading. Full-length blots are presented in Supplementary Figure S2. (**E**) Immunoblot analyses of cleaved caspase-3 derived from primary mandibular chondrocytes isolated from WT and *AhR*^−/−^ mice treated with or without AhR antagonist B[*a*]P (+ , oral gavage, 120 mg/kg). Staining of β-actin primary antibody was detected as a loading control. Full-length blots are presented in Supplementary Figure S2.

A number of genes which regulate metabolism are activated by the AhR, including genes encoding Cyp1 enzymes. In B[*a*]P-administered WT mice, higher levels of *Cyp1a1* mRNA and Cyp1a1 protein were detected (Fig. 3A,B). Immunohistochemical analysis further showed that B[*a*]P treatment of *AhR*^−/−^ mice did not affect condyle expression of Cyp1a1 (Fig. 3C). Thus, exposure to Ahr agonist B[*a*]P only significantly increased condyle levels of Cyp1a1, AhR and caspase-3 in WT mice (Fig. 3C). The active forms of caspase-3 and caspase-9 were found to be markedly upregulated after B[*a*]P treatment in chondrocyte progenitor cell line ATDC5 by immune blot analysis in a time-dependent manner (Fig. 3D). Furthermore, the accelerated expressions of cleaved caspase-3 were observed in the isolated mandibular chondrocytes of B[*a*]P-treated WT mice while activities of caspase-3 in those from B[*a*]P administered *AhR*^−/−^ mice was no change (Fig. 3E). These results might suggest that AhR/B[*a*]P signaling contributes to the Cyp1a1 expression and apoptotic molecules of mandibular chondrocytes via caspase signaling cascades.

### B[*a*]P affects mandibular condylar cartilage and expression of proteoglycan and aggrecan

**Figure 4:**
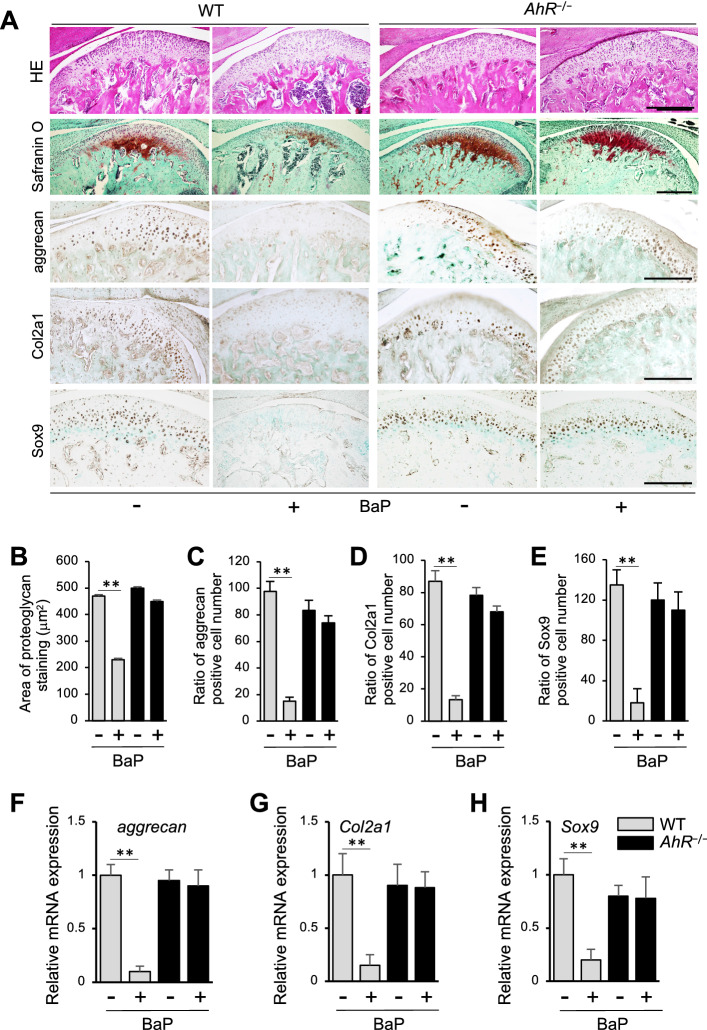
B[*a*]P inhibits chondrogenesis and chondrocyte differentiation. (**A**) Representative sections of mandibular cartilage from WT and *AhR*^−/−^ mice treated with or without B[*a*]P (+ , oral gavage, 120 mg/kg) which were stained with hematoxylin and eosin (HE), Safranin O and also subjected to aggrecan and Col2a1 IHC staining. Scale bar = 300 μm. (**B**) Area of proteoglycan staining in mandibular condyle cartilage from WT and *AhR*^−/−^ mice treated with corn oil only or B[*a*]P by oral gavage. (**C**–**E**) Numbers of cells expressing aggrecan (**C**), Col2a1 (**D**), or Sox9 (**E**) were counted in the posterior thirds or central of the mandibular condylar cartilages obtained from *AhR*^−/−^ and WT control mice treated with B[*a*]P were compared. (**F**–**H**) Following oral administrations of AhR ligand B[*a*]P (120 mg/kg) to WT and *AhR*^−/−^ mice, mRNA levels of *aggrecan* (**F**), *Col2a1* (**G**), and *Sox9* (**H**) were detected by real-time PCR. **p < 0.01.

HE specimens revealed subchondral bone loss and irregularities of chondrocyte alignment in the condylar cartilage layers in the B[*a*]P-treated WT mice while *AhR*^−/−^ samples showed no obvious histomorphological differences after B[*a*]P administration (Fig. 4A). OA-like degenerated lesions, including irregularities of chondrocyte alignment and extensive hyalinization in the condylar cartilage layers of the groups. Safranin O staining of proteoglycans in mandibular condylar cartilage from B[*a*]P-treated WT and *AhR*^−/−^ mice was performed. In control mice treated with corn oil, an abundance of proteoglycans was observed in the hypertrophic layer of the extracellular matrix (Fig. 4A,B). In contrast, the extent of proteoglycan staining in cartilage from B[*a*]P-treated WT mice was reduced. Moreover, fibrocartilage at the surface layer of the mandibular condylar cartilage was intact despite degradation of the mandibular condylar cartilage (Fig. 4A,B). For the B[*a*]P-treated *AhR*^−/−^ samples, Safranin O staining showed no obvious histomorphological differences (Fig. 4A,B). Meanwhile, IHC assays of articular cartilage resected from B[*a*]P-administered WT mice showed loss of aggrecan, Col2a1 and Sox9 protein expression. In *AhR*^−/−^ mice, there were no obvious histomorphological changes in articular cartilage after B[*a*]P treatment (Fig. 4A,C–E). Assays of mRNA levels of *aggrecan*, *Col2a1*, and *Sox 9* were also lower in mandibular condyles of B[*a*]P-administered WT mice (Fig. 4F–H). In contrast, these levels were unaffected in mandibular condyles of the *AhR*^−/−^ mice (Fig. 4F–H).

### Protective role of FICZ on mandibular cartilage damage and subchondral bone loss in experimental murine TMJ-OA

**Figure 5:**
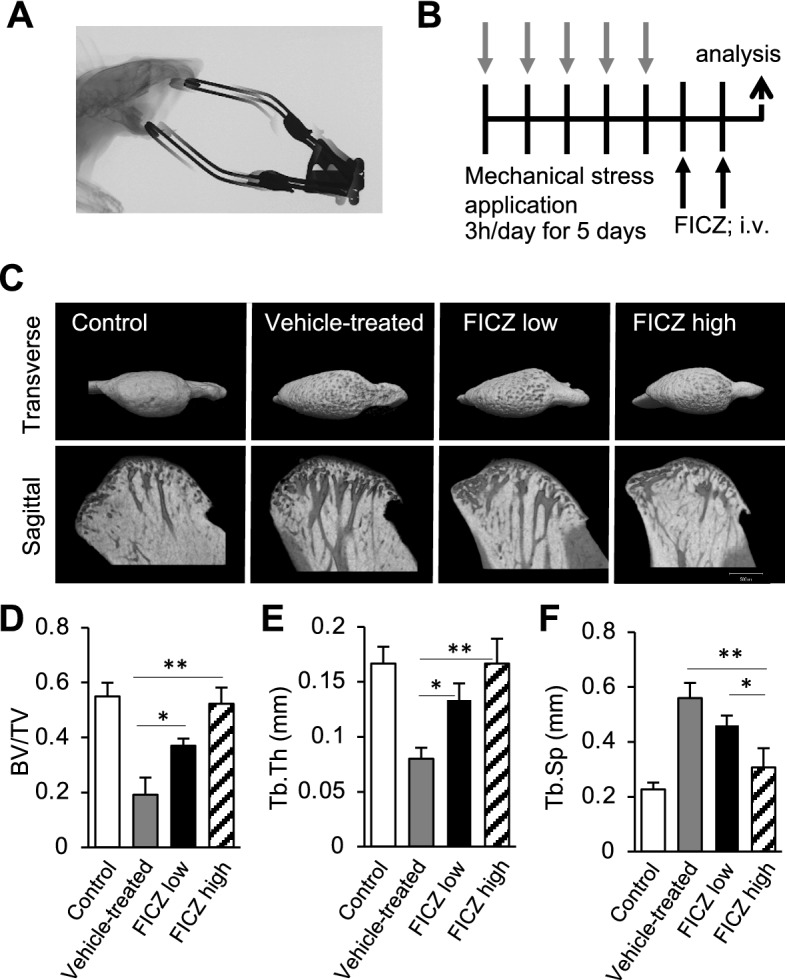
Mandibular subchondral bone volume and architecture are restored in FICZ-treated TMJ-OA mice. (**A**) A jaw opening device was applied to interincisal teeth of mice to achieve a maximal open posture. (**B**) Protocols and Experimental design. Mechanical daily overloadings were equally applied to bilateral TMJs with forced mouth-opening performed for 3 h each day. Charcoal or black arrows represent mechanical stress and administration with vehicle or FICZ, respectively. TRAP staining, micro-CT, TUNEL staining, and immunostaining analyses were performed according to protocol. (**C**) Representative transverse and sagittal view sections from micro-CT 3D scans of mandibular condyle sections from control, vehicle, and FICZ-treated TMJ-OA mice (FICZ low: 100 μg/kg, FICZ high: 100 mg/kg) are respectively shown. Scale white bar = 500 μm. (**D**–**F**) Values for the ratio between BV/TV (**D**), Tb.Th (**E**), and then Tb.Sp (**F**) are presented. (n = 7 per each group). **p < 0.01; *p < 0.05.

**Figure 6:**
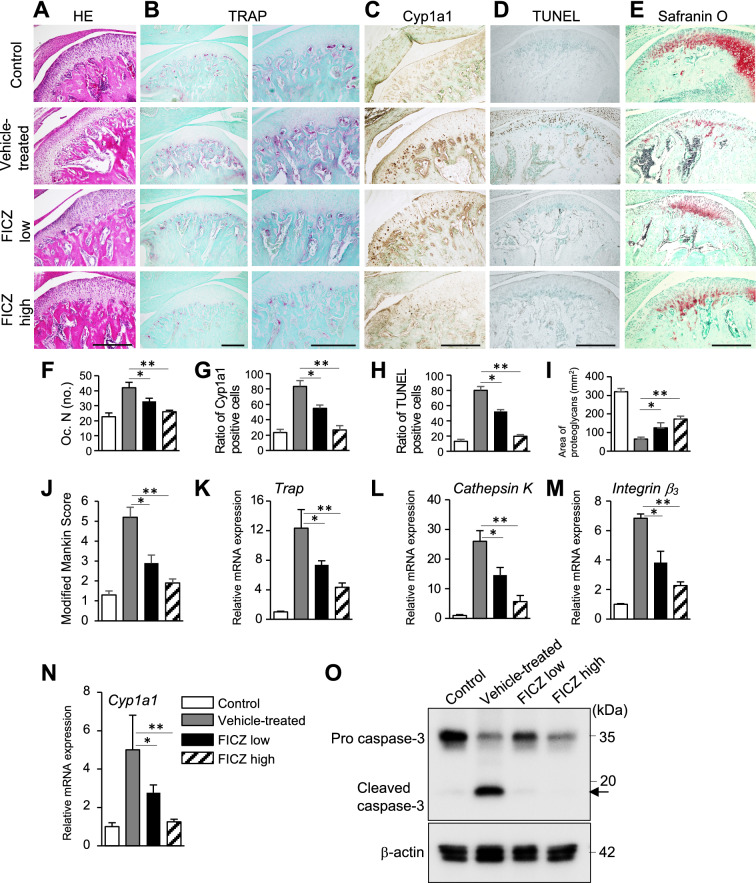
Protective role of FICZ on mandibular subchondral bone loss in experimental murine TMJ-OA. Representative sections of mandibular condylar cartilage from the control C57BL/6 WT mice and each of three-experimental treatment groups using TMJ-OA murine model (FICZ low: 100 μg/kg, FICZ high: 100 mg/kg vehicle-treated as control). (**A**) HE staining and (**B**) TRAP staining to examine histologic features of condylar cartilage. (**C**) IHC staining of Cyp1a1. (**D)** TUNEL staining. (**E)** Safranin O staining. Scale bar = 300 μm. (**F**) Quantitative analysis of TRAP^+^ osteoclastic (Oc.) cells/mm bone perimeter in subchondral bone. **(G)** Number of Cyp1a1^+^ cells. (**H**) TUNEL^+^ cells. (**I**) Area of proteoglycan staining. (**J**) Pathologic grading according to the modified-Mankin scores. (**K**–**N**) Detection of *Trap* (**J**), *Cathepsin K* (**K**), *Integrin β*_3_ (**L**), and *Cyp1a1* (**M**) mRNA levels. (**O**) Immunoblot analysis of cleaved and active form caspase-3 in primary cultured immature mandibular chondrocytes isolated from WT control mice and TMJ-OA mice administered with or without FICZ (vehicle-treated, FICZ low: 100 μg/kg, FICZ high: 100 mg/kg). Staining of β-actin was performed as a loading control. Full-length blots are presented in Supplementary Figure S2.

To establish a murine model of TMJ-OA, mechanical stress were applied to the TMJs of WT C57BL/6 mice. Briefly, a jaw maximal opening device was loaded to the interincisal-teeth holding the mandible in a maximal open posture (Fig. 5A)^15^. The TMJ-OA model was established after severe destruction of the subchondral bone was achieved. The mice then received an intravenous injection of FICZ dissolved in DMSO, or DMSO alone, twice a week (Fig. 5B). When three-dimensional (3D) reconstructions were generated of the subchondral bones in both treatment groups, severe destruction was observed in the TMJ-OA mice receiving DMSO alone (Fig. 5C–F). In contrast, destruction was prevented in a dose-dependent manner with FICZ administration (Fig. 5C–F). When TMJ sections from control WT mice were stained with HE, the articular cartilage exhibited a smooth surface and normal cellularity. In contrast, staining of the joints from the TMJ-OA mice revealed OA-like degenerated lesions, including irregularities of chondrocyte alignment in the condylar cartilage layers and subchondral bone loss. However, these lesions were attenuated in a FICZ dose-dependent manner (Fig. 6A). TRAP staining showed osteoclasts around the subchondral bone in the vehicle-treated TMJ-OA group. Meanwhile, the reduction in the numbers/activities of osteoclasts was observed a dose-dependently in two FICZ treatment group (Fig. 6B). When expression of Cyp1a1 was detected, the level was significantly higher in mandibular condyles from the vehicle-treated TMJ-OA group, while lower levels Cyp1a1 were observed dose dependently in the mandibular condyles from both groups of FICZ-treated TMJ-OA mice (Fig. 6C). To analyze whether normal or abnormal chondrocytes undergoing apoptotic cells were induced in degraded zones such as cartilage and subchondral bone layer, TUNEL staining was performed. Significant increase in the numbers of TUNEL^+^ cell were detected in the vehicle-treated TMJ-OA group compared to fewer TUNEL^+^ cells in the FICZ-treated TMJ-OA groups. The latter was associated with a protective effect against osteoclast-mediated subchondral bone resorption and cartilage degradation (Fig. 6D–J). Further characterization of condyles from the FICZ-treated TMJ-OA mice showed that mRNA expression levels of *Cathepsin K*, *Trap,* and *Integrin β*_3_ were markedly downregulated, as well as *Cyp1a1* expression (Fig. 6K–N). To further analyze and confirm the accelerated apoptosis that were observed in some layers of mandibular condyles in TMJ-OA, cleavage of caspase-3 was analyzed after the FICZ treatment. FICZ treatment dramatically repress the cleaved caspase-3 expression in the isolated mandibular chondrocytes in a dose dependent manner while active form of caspase-3 was upregulated in the vehicle-treated TMJ-OA group (Fig. 6O).

### FICZ disrupts actin rings to inhibit bone-resorption activity of osteoclasts

**Figure 7:**
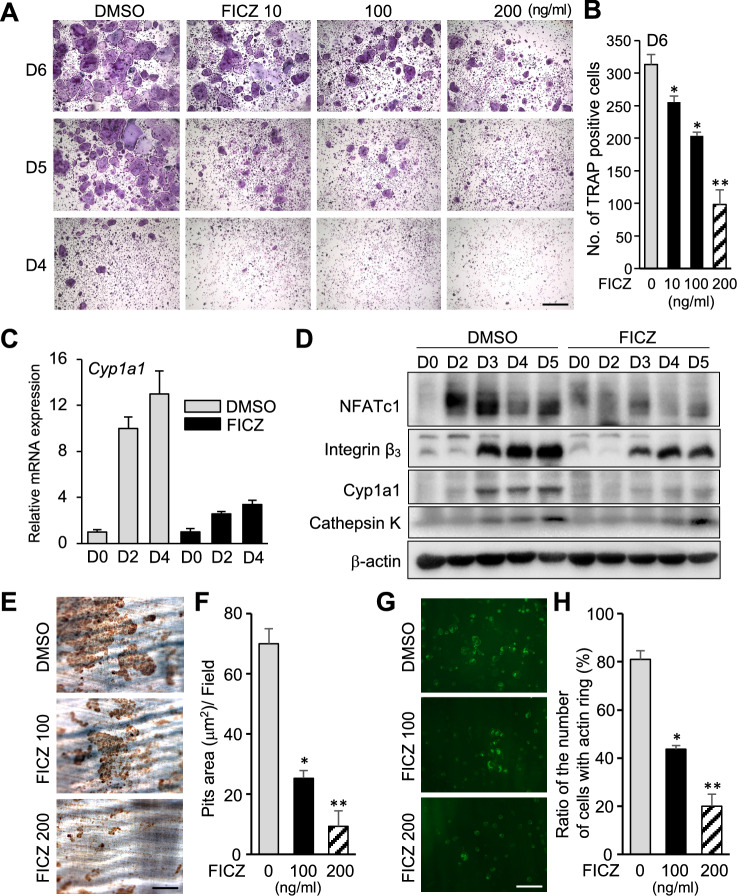
RANKL-mediated osteoclastogenesis is inhibited by FICZ. (**A**) BMMs stained with TRAP after being cultured with FICZ (10, 100, 200 ng/mL) during osteoclast differentiation stimulated by GST-RANKL (50 ng/mL) and recombinant mouse M-CSF (20 ng/mL) (days 4–6). (**B**) Quantification of TRAP^+^ multinucleated-osteoclasts in ten high-powered random fields per culture from each of four mice. Data represent the results of five independent experiments. (**C**) Detection of *Cyp1a1* mRNA in RANKL-stimulated BMMs cultures from WT mice treated with or without FICZ (200 ng/mL). (**D**) Western blot detection of NFATc1, integrin β_3_, Cyp1a1, and Cathepsin K at various time points of RANKL-stimulated BMMs cultures with or without FICZ (200 ng/mL) treatment until 5 days. Analysis by β-actin primary antibody was performed as a loading positive control. Full-length blots are presented in Supplementary Figure S2. (**E**) After osteoclasts were treated with FICZ (100, 200 ng/mL), bone resorbing activity was assayed. Mature osteoclasts were differentiated on the equal cut bone slices with 20 ng/mL recombinant M-CSF and 50 ng/mL GST-RANKL and then were exposed to FICZ (100, 200 ng/mL) until 6 days. A relative amount of resorbed area at each concentration of FICZ is presented (**F**). (**G**) Immunofluorescent and microscopic detection of F-actin in osteoclasts stimulated in the presence of FICZ (100, 200 ng/mL). The accompanying bar graph presents the ratio of the number of cells having an actin ring (**H**). Scale white bar = 100 μm; **p < 0.01; *p < 0.05.

To further investigate the effect of the relationship between FICZ and AhR axis on osteoclasts, WT BMMs were treated with FICZ, an endogenous AhR ligand. Formation of TRAP^+^ cells in cultures of WT BMMs was attenuated in a FICZ dose-dependent manner (Fig. 7A,B). In contrast, treatment with RANKL dramatically increased *Cyp1a1* expression in DMSO-treated WT BMMs, yet not in FICZ-treated cells (Fig. 7C). Protein levels of Cyp1a1, Cathepsin K, Integrin β_3_, and NFATc1 expressions were also decreased following FICZ treatment by immunoblot analysis (Fig. 7D). Cytoskeletal reorganizations, representatively actin ring formation, is closely related to the bone-resorbing activities of activated osteoclasts^24^. When RANKL-stimulated pits-formation assays were performed, a dose-dependent effect of FICZ treatment was observed. For example, the bone slices-resorbing activities of activated osteoclasts was in part inhibited in 100 ng/mL FICZ, or was nearly inhibited in 200 ng/mL FICZ (Fig. 7E,F). Correspondingly, actin rings were essentially disrupted within 10 h of FICZ addition (Fig. 7G,H). Taken together, these results might indicate that FICZ-mediated suppression of the osteoclastic bone resorption activity may involve disruption of actin rings.

### FICZ positively regulates osteoblastogenesis

FICZ regulates bone formation activities because the calcifications and differentiations of MC3T3 E1 cells into the bone-forming osteoblastic cells were accelerated by FICZ treatment (Supplemental Fig. S1A,B). Furthermore, the key osteobastic gene expressions of *Alpl*, *Osteocalcin*, and *Col1a1* mRNAs were upregulated in the FICZ- stimulated MC3T3 E1 cells (Supplemental Fig. S1C). These results are largely supported by the findings of in vivo imaging analyses of the bones and minerals apposition rates determined by tetracyclines and calcein-double fluorescence labelling, which also showed significant acceleration in minerals apposition or bone formations rate in FICZ-treated mice (Supplemental Fig. S1D–F).

## Discussion

Skeletal healing and bone mass in individuals with a history of smoking have been found to be adversely compromised, independent of gender. In particular, nicotine has been found to inhibit distraction osteogenesis^25^, spinal fusion^26^, and fracture healing in rabbits^27^. Traditionally, smoke-induced bone fractures or osteoporosis have been attributed to impaired the osteoblastic bones and minerals formation. However, conflicting reports regarding the role of tobacco-smoke carcinogens on bone resorption have been published^28,29^. Dioxins are a component of cigarette smoke and exhibit a capacity to inhibit osteoblast formation and differentiation^30–32^. Moreover, it has been reported that the low dose of the AhR agonist concentrations such as 2,3,7,8-tetrachlorodibenzo-p-dioxin (TCDD) or B[*a*]P accelerates osteoclastogenesis and bone mineral resorptions, while low rates of osteoclastogenesis are observed in *AhR*^−/−^ mice^23^. In our own recent study, we demonstrated that the AhR-c-Fos signaling axis in osteoclasts helps regulate fracture healing^8^. These findings are consistent with the hyperresorption that is observed in smokers^28,29^, and the ~ 60% increase in bone resorption that characterizes mice overexpressing constitutively activated *AhR*^33^. In mice exposed to smoke, delays in chondrogenesis have been associated with tibial fractures^34^. Consequently, we hypothesize that B[*a*]P, one of the major components of cigarette-smoke, impairs bone metabolism in mandibular condyles via AhR ligands. It has been reported that AhR ligands mediate toxic effects on various types of tissues, including hard palate tissue and tooth^35,36^. However, the effects of the AhR pathway on mandibular condyle tissue have remained unclear. In particular, the effects of B[*a*]P and FICZ on mandibular bone metabolism or TMJ-OA have not been investigated. Therefore, to the best of our knowledge, our present research is the first and novel to report B[*a*]P-mediated effects on mandibular subchondral bone, which is a site of tissue healing distinct from the lung. To evaluate biological effects of cigarette smoke on cultured cells, B[*a*]P containing in cigarette smoke have been generally used. However, use of B[*a*]P or smoke extracts may not completely mimic in vivo smoking situations. Overall, we observed that the AhR-targeted gene, *Cyp1a1*, is upregulated in those from mandibles of mice following exposure to B[*a*]P, which implies those activation in AhR signaling may be involved.

Previously, it was demonstrated that α-naphthoflavone inhibits Cyp1a1 activity and significantly reduces B[*a*]P-induced apoptosis^37^. In contrast, 2,3′,4,5′-tetramethoxystilbene and pyrene, specific inhibitors of Cyp1b1, do not suppress B[*a*]P-induced apoptosis. Kim et al. also reported that Cyp1a1, and not Cyp1b1, is involved in mediating B[*a*]P-induced activation of cleaved caspase-3 in human RL95-2 endometrial cancer cells and Hepa1c1c7 cells^38^. In our present studies, a significant increase of *Cyp1a1* mRNA was detected in the mandibular condyles from B[*a*]P-administered-WT mice. In brief, these data indicate that B[*a*]P-liganded AhR may activate caspase-3 cascade via the genomic pathways which involves rapid transcriptional regulations of *Cyp1a1* mRNA in mandibular chondrocytes. An alternative pathway to B[*a*]P-induced DNA damage involves signaling-crosstalk between AhR axis and other signaling pathways which regulates the differentiation of osteoclasts and chondrocytes. Accumulating evidences support an essential role for hypoxia-inducible factor (HIF) signaling in osteoclastogenesis and chondrogenesis^39–41^. Moreover, the transcriptional activities of HIF-1 and AhR are ARNT dependent. Correspondingly, several studies have demonstrated that inhibitory crosstalk takes place between these two signaling pathways^42–44^. Thus, it is possible that this same cross talk affects osteoclast and chondrocyte differentiation as well. Nicotine, an exogenous stimulator of the cholinergic system, is also important component of cigarette smoke. Several studies reported the role of cholinergic system in the bone homeostasis in the context of osteoporosis and osteoarthritis^45,46^. Acetylcholine or exogenous activation via nicotine or muscarine, stimulates osteoblast proliferation and osteoclast apoptosis^45^. Acetylcholinesterase (AChE), an enzyme catalyzing the degradation of acetylcholine, plays an important role in the cholinergic regulation by terminating the action of acetylcholine. It has recently been reported that the expression of AChE can also be found in non-neuronal tissues like the immune system and bone tissue^46^. After induction of bone loss in mice using RANKL, the AChE inhibitor donepezil could resucue the RANKL-induced bone loss. During osteoclastogenesis of bone marrow macrophages an upregulation of the AChE expression has been observed. Moreover, genetic knockdown of AChE via siRNA suppressed RANKL-induced osteoclast differentiation^46,47^. These results suggest that the signaling interaction between AhR and AChE might be a potential therapeutic target for bone disease like osteoporosis and TMJ-OA.

The TMJ-OA experimental model that was established in the present study with mechanical stress applied to the mandibular condyle is consistent with that used in other studies^15,48–52^. This model is characterized by chondrocyte alignment irregularities in the layers of mandibular condylar cartilage, marked depletion of proteoglycans, and subchondral bone destructions, and OA-like degenerated lesions. It has been recognized that OA is not only a problem of cartilage damage but also a whole joint disorder^3,4^. In particular, articular cartilage and subchondral bone function as a unit to maintain the structural and functional integrity of the joint^4,9^. The animal models to recapitulate the disturbance of subchondral bone as well as cartilage degeneration are needed to better understand the cartilage bone unit in the pathogenesis of TMJ-OA. The results obtained in the present study with this model are also consistent with those reported for models of early TMJ-OA induced with surgical joint manipulations^53,54^, local deliveries or applications of chemicals^55,56^, and gene deletions or modification^57,58^. The origin of TMJ-OA is primarily non inflammatory, unlike synovitis and rheumatoid arthritis. The pathology of TMJ-OA involves abrasion and deterioration of the mandibular articular cartilage, as well as local turnover and thickening of the underlying bone remodeling. Moreover, those changes are often involved in secondary inflammatory reactions. In the TMJ, excessive mechanical stress is one of the key factors in the induction of degradating cartilages in the mandibular condylar. Consequently, use of forced mouth opening is successful in establishing an experimental TMJ-OA model mouse to evaluate the initialization and progression toward TMJ-OA.

In these current studies, administration of the AhR endogenous ligand, FICZ, significantly attenuated bone loss in the mandibular subchondral bone and the inflammatory response in our TMJ-OA model. Subchondral bone remodelling occurred with angiogenesis prior to microscopic changes in articular cartilage on the posterior tibia in the anterior cruciate ligament transection (ACLT) mouse model^59^. Our result consistent with the previous reports that the subchondral remodeling reflects the early stage of OA^9^. In addition to routine bone parameters, evaluation of the porosity in the subchondral bone might be an emerging indicator in the early stage of TMJ-OA. The in-depth understanding of subchondral bone disturbance in TMJ-OA will lead to a paradigm shift in therapies for TMJ-OA. Similar results also have been obtained in other studies of FICZ. For example, in a murine model about ligature-induced periodontitis, FICZ treatment reduced the inflammatory response^60^. Similarly, FICZ downregulated epithelial-derived IL-7 expressions and repressed inflammation in the gastro-intestinal tract of mice with DSS-induced colitis^61^, and has inhibited imiquimod-induced skin inflammation^62^. However, it has been observed that AhR ligands are not universally applicable for controlling inflammation. For example, while TCDD (also known as another family of smoke contaminants as dioxin) exhibits immunomodulatory properties, it is the most toxic among the AhR ligands^63^. Inhibition of osteoclast formation by 3-methylcholanthrene (3MC) containing in cigarette smoke is evident at very low concentrations (10^–9^ M), whereas inhibition of osteoblast formation occurs at higher concentrations (10^–7^ M), suggesting that 3MC has a stronger inhibitory effect on osteoclastogenesis than osteoblast formation^64,65^. Further research also suggests the preferential targeting of osteoclasts by 3MC and other AhR ligands by examining the downstream effects of the AhR signaling. Another AhR-ligands, kynurenines, are secreted from the human tumors to promote production of regulatory T cells (Tregs) in order to mediate potential autoimmune issues^66^. The present results indicate that FICZ is able to mitigate TMJ-OA. To better elucidate the mechanisms mediating regulation of inflammation by AhR signaling, we performed in vitro cell culture assays with BMMs. Following treatment with FICZ, RANKL-induced formation of osteoclasts was found to be inhibited. FICZ treatment also reduced RANKL-induced expression of Cyp1a1, as well as the osteoclastic markers, integrin β_3_, NFATc1, and cathepsin K. Huai et al.^67^ previously demonstrated that pre-treatment with increasing concentrations of FICZ reduced expression of NLRP3 in mouse macrophages stimulated with lipopolysaccharide and inflammasomes. When TMJs were compared between mice with and without FICZ treatment, the former exhibited a dose-dependent attenuation of cartilage degradation within the hypertrophic layer of the condylar cartilage. Further research is essential to better understand the role of these changes in relation to chondroprotection and homeostasis of the ECM in cartilage as well as the molecular mechanism of osteoblastogenesis. Survival of OA chondrocytes also requires further study.

In conclusion, we present data supporting AhR expression in mandibular condyles and activation of AhR signaling upon binding of B[*a*]P. Furthermore, in the presence of B[*a*]P, osteoclast activity as well as the expression of aggrecan, Col2a1 and Sox9 are altered in the subchondral bone and mandibular chondrocytes. It is possible that overactivation of the AhR pathway induces disordered bone remodeling and inappropriate bone turnover in mandibular condyles. Based on the data we obtained from qPCR analysis, radiographs of mandibular condyles, and histological assays, the AhR endogenous ligand, FICZ, provides protection from inflammatory responses and prevents bone loss in mandibular subchondral bone in TMJ-OA. It is anticipated that these findings will facilitate the development of a new therapeutic approach for AhR-initiated inflammatory diseases, in combination with further evidences of a mechanistic details of AhR signaling in mandibular condyles.

## Supplementary Information

Supplementary Informations.
