# Supplementary material for: Roles for B[a]P and FICZ in subchondral bone metabolism and experimental temporomandibular joint osteoarthritis via the AhR/Cyp1a1 signaling axis
Source: Sci Rep. 2021 Jul 21;11:14927. doi: 10.1038/s41598-021-94470-4 (PMC8295293; doi:10.1038/s41598-021-94470-4)
Supplement: Supplementary file 1 — Supplementary Informations. [file 41598_2021_94470_MOESM1_ESM.pdf]

Supplemental Figure S1. Effect of FICZ on osteoblastogenesis. MC3T3 E1 cells were cultured with osteogenic media in the presence of FICZ during up to 21 days. After fixing by methanol or 70% ethanol, cells were stained with Alizarin Red (**A**) and ALP (**B**). (**C**) Total RNA were purified from MC3T3 E1 cells that were stimulated with (bold line) or without (dot line) FICZ (200 ng/ml). (**D**) Fluorescent double labels of newly formed bones in vehicle-treated or FICZ treated mice labeled with tetracycline (yellow) and calcein (green) on day 0 and 5 respectively and then sacrificed on day 7. Scale bar = 50 μm. Quantification of BFR; bone formation rate (**E**) and MAR; mineral apposition rate (**F**) of mice. $^{**}p < 0.01$, $^{*}p < 0.05$.

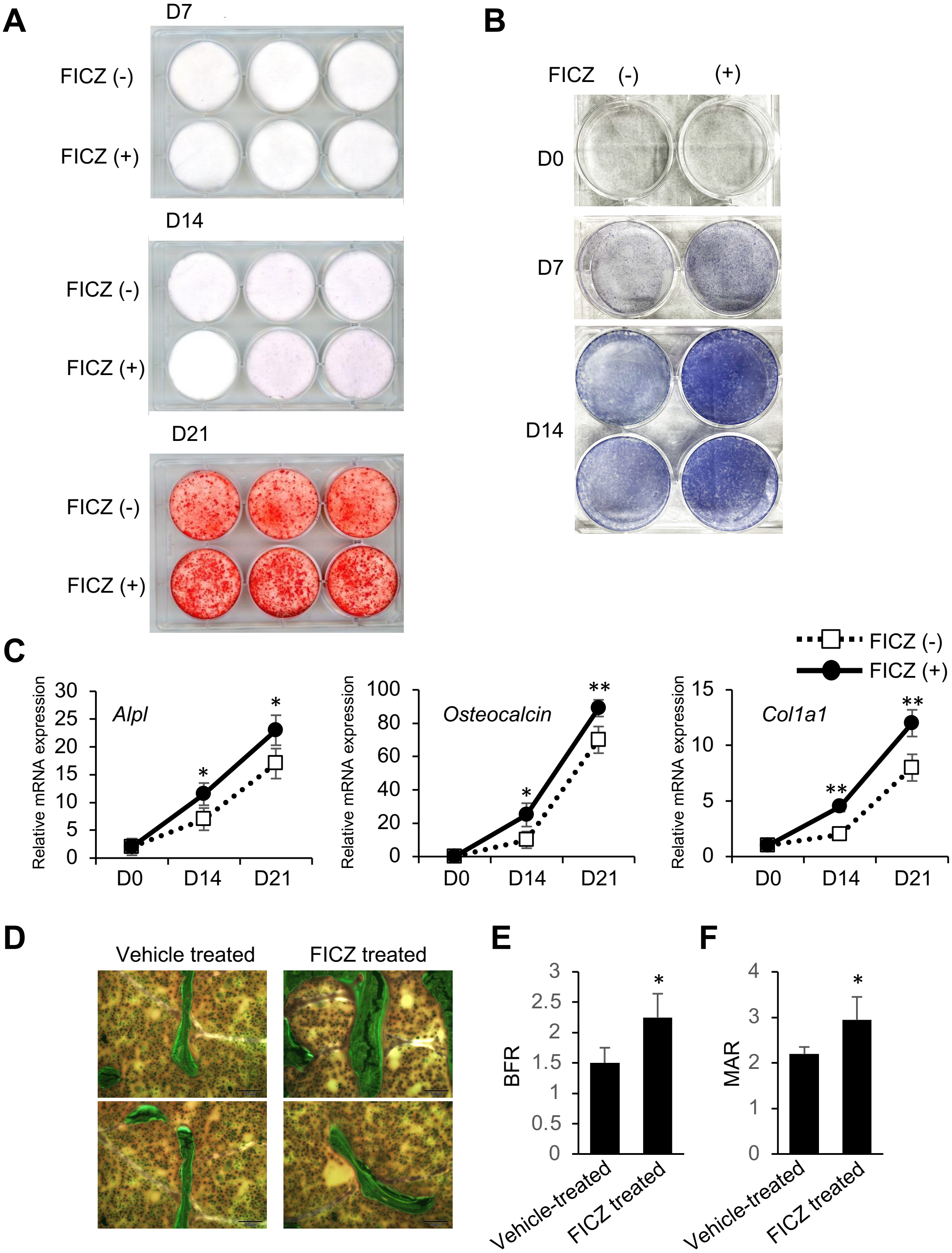

Supplemental Figure S2. Uncropped western blot images. The red sections mark blot and gel results shown in the indicated figures.

Figure 3B and its originals

Cyp1a1 β-actin

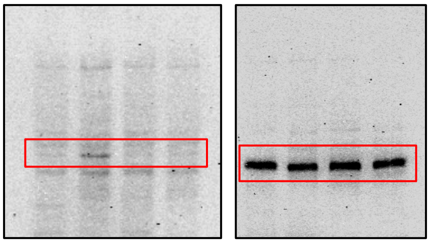

Figure 3D and its originals

Cleaved Caspase-3

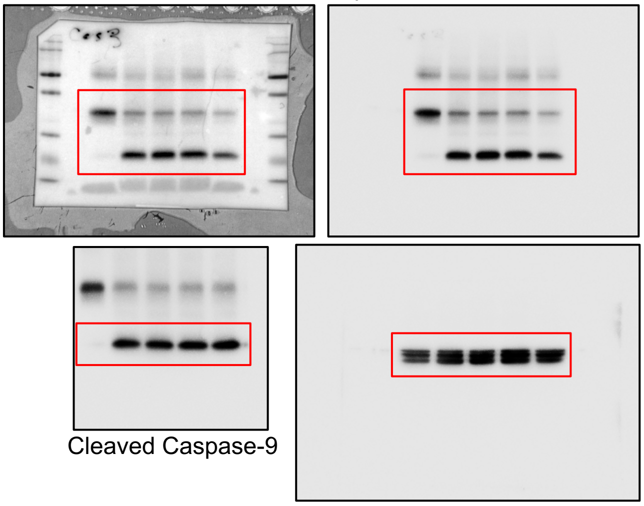

β-actin

Figure 3E and its originals

Cleaved Caspase-3

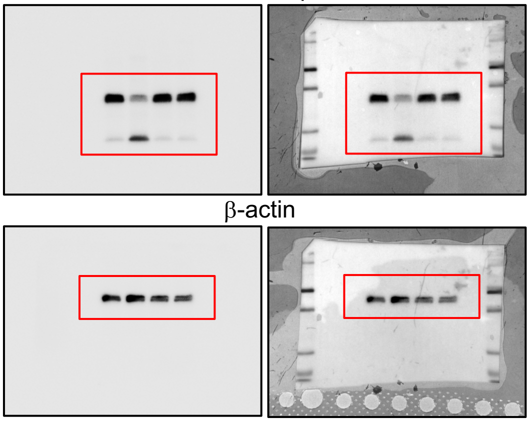

Figure 6O and its originals

Cleaved Caspase-3

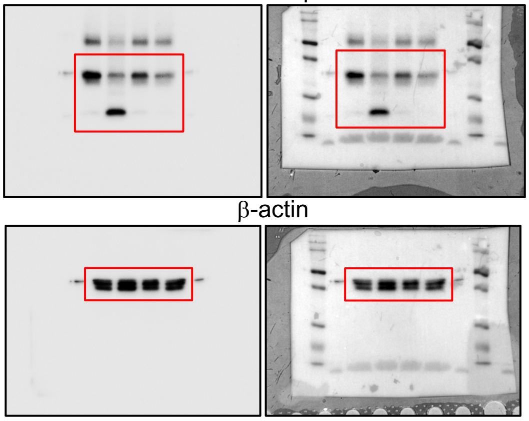

Figure 7D and its originals

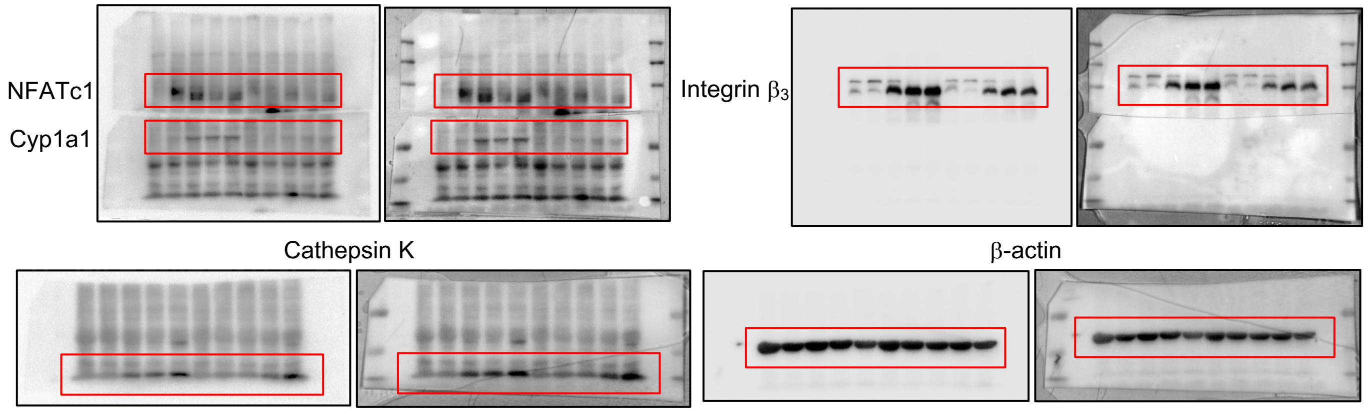

Supplemental Table S1. Sequences of the primers used in RT-PCR assays.

Gene	Sense	Antisense
Cyp1a1	5’-CATCACAGACAGCCTCATTGAGC-3’	5’- CTCCACGAGATAGCAGTTGTGAC -3’
Trap	5’-CAGCTCCCTAGAAGATGGATTCAT-3’	5’-GTCAGGAGTGGGAGCCATATG-3’
Cathepsin K	5’-AGGCAGCTAAATGCAGAGGGTACA-3	5’-AGCTTGCATCGATGGACACAGAGA-3’
Integrin β_3	5’-TTCGACTACGGCCAGATGATT-3’	5’-GGAGAAAGACAGGTCCATCAAGT-3’
aggrecan	5’-CAGGCTATGAGCAGTGTGATGC -3’	5’-GCTGCTGTCTTTGTCACCCACA -3’
Col2a1	5’-GCTGGTGAAGAAGGCAAACGAG -3’	5’-CCATCTTGACCTGGGAATCCAC -3’
Sox9	5’-AGTACCCGCATCTGCACAAC -3’	5’-ACGAAGGGTCTCTTCTCGCT-3’
Alpl	5’-AACCCAGACACAAGCATTCC -3’	5’-GCCTTTGAGGTTTTTGGTCA -3’
Osteocalcin	5’-CAGCGGCCCTGAGTCTGA -3’	5’-GCCGGAGTCTGTTCACTACCTTA -3’
Col1a1	5’-GAGCGGAGAGTACTGGATCG -3’	5’-GTTAGGGCTGATGTACCAGT -3’
GAPDH	5’-AGGTCGGTGTGAACGGATTTG -3’	5’-TGTAGACCATGTAGTTGAGGTCA-3’
